# Metabolic Syndrome: A Strange Companion of Atrial Fibrillation; A Blessing in Disguise from the Neuropsychiatric Point of View

**DOI:** 10.3390/biomedicines11072012

**Published:** 2023-07-17

**Authors:** Ciprian Ilie Rosca, Daniel Florin Lighezan, Daniel-Dumitru Nisulescu, Abhinav Sharma, Marioara Nicula Neagu, Daciana Nistor, Doina Georgescu, Nilima Rajpal Kundnani

**Affiliations:** 1 Department of Internal Medicine I—Medical Semiotics I, Centre for Advanced Research in Cardiovascular Pathology and Haemostasis, “Victor Babeș” University of Medicine and Pharmacy, Eftimie Murgu Sq. no. 2, 300041 Timișoara, Romania; rosca.ciprian@umft.ro (C.I.R.); doina.georgescu@umft.ro (D.G.); 2 General Medicine Faculty, “Vasile Goldis” West University, 473223 Arad, Romania; 3 Department of Cardiology—Internal Medicine and Ambulatory Care, Prevention and Cardiovascular Recovery, “Victor Babeș” University of Medicine and Pharmacy, Eftimie Murgu Sq. no. 2, 3000041 Timișoara, Romania; sharma.abhinav@umft.ro (A.S.); knilima@umft.ro (N.R.K.); 4 Faculty of Bioengineering of Animal Resources, Discipline of Physiology, University of Life Sciences “King Mihai I” from Timisoara, 300645 Timisoara, Romania; mnicula@animalsci-tm.ro; 5 Department of Functional Sciences, Physiology, Centre of Imuno-Physiology and Biotechnologies (CIFBIOTEH), Victor Babes University of Medicine and Pharmacy, 300041 Timisoara, Romania; daciana_nistor@yahoo.com; 6 Centre for Gene and Cellular Therapies in Cancer, 3000723 Timisoara, Romania

**Keywords:** atrial fibrillation, metabolic syndrome, stroke, dementia, cognitive impairment

## Abstract

**Background:** The concept of metabolic syndrome (MetSy) brings together components that individually represent a risk factor for cardiovascular diseases, which over time can prove to be more harmful if a combined effect of these is exhibited. **Method**: A single-centre retrospective study in an academic medical unit was conducted. We analysed the link between the MetSy and the occurrence of neuropsychic complications among atrial fibrillation (AF) patients. We sifted through the files of the patients admitted during 2015–2016 to the Municipal Emergency University Hospital Timisoara, Romania, with the diagnosis of AF. We divided these AF patients into two groups: the first group comprised patients with atrial fibrillation and MetSy (267 patients), while the second group comprised AF patients without MetSy (843 patients). We analysed the occurrence of neuropsychic changes (stroke, Parkinson’s disease, dementia, cognitive impairment, and silent lacunar infarction) among the two groups. **Results**: Cognitive impairment (*p*-value = 0.0081) and dementia (*p*-value < 0.0001) were less frequent in patients with AF and MetSy than in those with AF without MetSy. Regarding the presence of stroke and Parkinson’s disease (PD), we could not demonstrate the existence of any statistically significant difference between the two groups. Using logistic regression (enter test), we found that MetSy might have a protective effect (OR = 0.4040, 95% CI [0.2132; 0.7654], *p*-value = 0.0054) for the occurrence of dementia in those patients. Furthermore, obesity was the only factor with a possible protective effect from all the constituents of the MetSy when analysed together (with a significance level of *p*-value = 0.0004 for the logistic regression). The protective effect of MetSy against stroke occurrence was supplementarily proven by a longer period of survival without stroke from the AF diagnosis (3.521 years, *p* = 0.0304) compared to patients with AF without MetSy (3.286 years to first stroke occurrence). **Conclusions**: Metabolic syndrome might offer protection against the occurrence of dementia among patients with AF, but no effect was noted when compared with the presence of stroke. Further studies on larger cohorts can help us reach a conclusion regarding the positive effects of the metabolic syndrome.

## 1. Introduction 

Despite the fact that the definition of metabolic syndrome (MetSy) does not include the presence of hyper-LDL-cholesterolemia, smoking, family medical history, age, or gender, nor is it as useful as the Framingham score for determining the total cardiovascular risk, it is extremely useful in current medical practise because it helps identify an important subgroup of patients. These patients exhibit cardiovascular complications in a similar manner from a pathophysiological point of view [1].

Atrial fibrillation (AF), with an increasing prevalence in the last 20 years [2,3,4], is the most common cardiac arrhythmia seen [2,4]. AF is still an important cause of neuropsychic complications (NPSC), even in patients with a proper therapeutic regimen.

Lone atrial fibrillation is a risk factor for developing NPSC, but when AF is present in patients with subsequent comorbidities, it increases the incidence of NPSC. Factors predisposing to the occurrence of AF, such as obesity and diabetes, are also the two constitutive factors of MetSy, and they also associate with an increased incidence of NPSC in the absence of AF and thus are of major importance in day-to-day practise. Actively combating them thus becomes a paramount step in the prevention of NPSC and its complications [5,6].

MetSy is an association based on the mechanisms of action of several cardiovascular risk factors: obesity or an increased abdominal circumference; hypertriglyceridemia; low serum high-density lipoprotein (HDL) cholesterol; the presence of arterial hypertension; type 2 diabetes mellitus (DM); or raised fasting plasma glucose [7].

The presence per se of the MetSy predisposes to a tripling of the risk of developing stroke [7], but it also increases the risk of mild cognitive impairment as well as dementia (especially vascular dementia), including the progression of mild cognitive impairment [8].

Even without diabetes mellitus, or MetSy, there is a strong correlation between elevated blood glucose levels and an increased risk of cognitive impairment or dementia [9].

Obesity is recognised as an important determinant of the occurrence of cardiovascular diseases, including AF and stroke, increasing cardiovascular mortality and morbidity both directly and indirectly [10]. However, there are studies that show an increased incidence of cognitive impairment in older male patients with lower weight and height [11].

Arterial hypertension predisposes to stroke both as a short-term complication and as a long-term complication [12]. It also predisposes in the long term to AF, dementia, and diabetes mellitus [12]. The control of blood pressure values is extremely useful in reducing the risk of dementia, stroke, and AF in patients with high blood pressure [6].

The involvement of MetSy in the occurrence of cognitive impairment is still unclear and controversial. There are studies that have shown that in elderly male patients, its presence is associated with a statistically significant lower risk of developing cognitive impairment compared to the general population [11]. On the other hand, MetSy seems to be responsible for the appearance of mild cognitive impairment manifested by decreased memory [13].

Mild cognitive impairment and Alzheimer’s disease have been shown to be associated with MetSy based on inflammatory changes in white matter in rats [14].

The existence of contradictory data regarding the involvement of cardiovascular risk factors in the occurrence of AF and stroke has imposed new notions known as paradoxes: “the obesity paradox”, “the hypercholesterolemia paradox”, and even “a hyper LDL-cholesterolemia paradox” [10,15,16].

The primary outcome of our study was to evaluate the impact of MetSy among AF patients in the presence of NPSC. The secondary outcome was to evaluate the contribution of each MetSy component to the NPSC.

## 2. Material and Methods

This retrospective study was conducted at the Municipal Emergency Hospital in Timisoara. We analysed the files of the patients admitted to the medical ward with the diagnosis of AF between 1 January 2015, and 31 December 2016. Before initiating the study, approvals from the Hospital management and the Ethics Committee of the “Victor Babes” University of Medicine and Pharmacy, Timisoara, were obtained.

The only inclusion criteria taken into account in our study was the presence of AF. There was no limitation regarding gender, age, ethnicity, socio-economic status, previous medications, or other comorbidities. We excluded from our study all the patients admitted without an AF diagnosis.

A total of 1111 AF cases were analysed for the presence of stroke, dementia, silent lacunar infarction, cognitive impairment, and PD after the onset of AF, including the presence of MetSy.

The presence of stroke, dementia, silent lacunar infarction, cognitive impairment, and PD was noted if the diagnosis was made by a neurologist, psychiatrist, or radiologist (in their current admission files or from previous medical evaluation records).

Only one patient was excluded after initial verification of the data due to incomplete records. We divided these 1110 patients into two groups. The first group consisted of patients with AF associated with MetSy (*n* = 267 patients), and Group 2 had AF but without MetSy (*n* = 843 patients).

AF was diagnosed using a 12-lead electrocardiographic recorder at admission [4]. The CHA_2_DS_2_-VASc score was calculated according to the guidelines and definition [4].

We defined MetSy according to the International Diabetes Federation (IDF) criteria. The association of central obesity (using abdominal circumference) or obesity with two of the following: serum triglyceride ≥ 150 mg/dL (or treatment for hypertriglyceridemia), decrease in HDL cholesterol value ≤ 40 mg/dL for men, respectively ≤50 mg/dL for women, increase in systolic BP ≥ 130 mmHg or diastolic BP ≥ 85 mmHg, and raised fasting plasma glucose (RFPG) ≥ 100 mg/dL or previous diagnosis of type 2 diabetes mellitus [7]. To define it in accordance with the IDF recommendations, the simultaneous presence of at least three of the following: arterial hypertension, diabetes, obesity, hypertriglyceridemia, or hypo HDL cholesterolemia, was noted.

The nutritional status was stratified according to body mass index (BMI) as overweight if the BMI was less than 29.9 kg/m^2^ but greater than 25 kg/m^2^, and obese if the BMI was greater than or equal to 30 kg/m^2^.

For the staging of chronic kidney disease (CKD), we calculated the glomerular filtration rate (GFR) using the 2012 CKD-EPI formula, and the staging was done using the KDIGO guidelines [17]. Hyperuricemia was defined as serum uric acid values greater than 6.0 mg/dL (the normal upper limit displayed by the hospital clinical laboratory) and noted.

Statistical analysis was performed using MedCalc Statistical Software version 20.015 (MedCalc Software Ltd, Ostend, Belgium) with a significant *p*-value < 0.05. We used descriptive statistics, figures, and tables to summarise our findings. Results for targeted variables were presented using descriptive statistics (mean, standard deviation, range, median, and associated interquartile range) for continuous data and counts with associated percentages for categorical data. The independent samples *t*-test was used to analyse differences in means for continuous variables, while differences between categorical variables were examined by the chi-squared test or Fischer’s exact test. Categorical data are presented as counts (percentages). Logistic regression analysis was considered to evaluate the odds of neurological diseases (stroke, dementia, and Parkinson’s disease). A survival analysis was made based on Kaplan-Meier curves.

## 3. Results

We divided our 1110 patients into two groups. Group 1: 267 patients with AF and MetSy (representing 24.1% of all study patients). Group 2: 843 patients with AF but without MetSy (representing 75.9% of all study patients). Details are presented in Table 1.

The two study groups were compared from a gender point of view. Still, regarding age, we found that the Group 1 patients were younger compared with the Group 2 patients, with a 3.7-year difference between them (*p*-value < 0.0001, 95% CI [2.3548; 5.0634]).

Even though hyperuricemia is not among the parameters included in the definition of MetSy, it was almost twice as frequently present in Group 1 patients (*p*-value < 0.0001). The MetSy was not found statistically significant for the presence of COPD, the presence of CKD above stage 3, or the place of residence.

**Figure 1:**
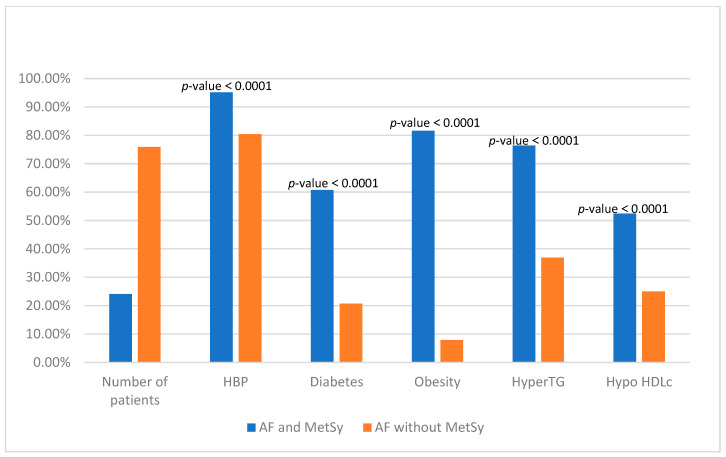
Presence of components of the metabolic syndrome among two groups. HBP—arterial hypertension; HyperTG—hypertriglyceridemia; Hypo HDLc—hypo high-density lipoprotein cholesterol; AF—Atrial Fibrillation; MetSy—metabolic syndrome; and p—statistically significance level.

The presence of MetSy components among the two groups is presented in Figure 1.

The mean CHA_2_DS_2_-VASc for both groups showed no statistical difference. In Group 1, it was 4.9549 (95% CI [4.8234; 5.0864]), and in Group 2, it was 5.1124 (95% CI [4.9049; 5.3198]).

The presence of stroke, dementia, Parkinson’s disease (PD), cognitive impairment, and silent lacunar infarction (SLI) was evaluated in both groups. Cognitive impairment (*p*-value = 0.0081) and dementia (*p*-value < 0.0001) were seen to be less present in patients with AF and MetSy than in patients with AF without MetSy. The statistical analysis for these parameters is presented in Table 2.

Considering the data presented above, a supplementary statistical analysis was done to find out which component is responsible for protection against dementia. Hence, using the logistic regression test, we found that MetSy has a protective role (OR = 0.4040, 95% CI [0.2132; 0.7654], *p*-value = 0.0054) for the occurrence of dementia in these patients.

Obesity is the only factor with a protective effect from all the constituents of the MetSy when they are analysed together (with a significance level of *p*-value = 0.0004 for the logistic regression) (Table 3).

In the same manner, we found that MetSy might have a protective effect regarding the presence of stroke in AF patients (*p*-value < 0.0001). Analysing the components of MetSy, we found that obesity (coefficient −0.70929, OR 0.4920, 95% CI [0.3625; 0.6677], *p*-value < 0.0001) and the male gender (coefficient −0.37708, OR 0.6859, 95% CI [0.5339; 0.8810], *p*-value = 0.0032) are the only factors that alone offer benefits in a statistically significant manner.

Knowing that each constituent element of MetSy is also a risk factor for the occurrence of NPSC, we analysed the impact of their individual presence in NPSC among the two groups.

DM does not represent a risk factor of greater importance in patients with MetSy than in those without MetSy. Comparing these patients, no statistical difference was detected in the occurrence of stroke (*p*-value = 0.1709), dementia (*p*-value = 0.0648), cognitive impairment (*p*-value = 0.6809), SLI (*p*-value = 0.1929), or PD (*p*-value = 0.3454). These data were obtained despite the fact that Group 1 diabetic patients presented with significantly more hypertriglyceridemia (*p*-value = 0.0009), HBP (*p*-value = 0.0005), obesity (*p*-value < 0.0001), and hyperuricemia (*p*-value = 0.0062).

Patients with low levels of serum HDL-cholesterol showed a statistically significant increased occurrence of stroke (*p*-value = 0.0006) and dementia (*p*-value = 0.0110), but not cognitive impairment (*p*-value = 0.0604), SLI (*p*-value = 0.3478), or Parkinson’s disease (*p*-value = 0.0834), respectively. Patients with low levels of serum HDL-cholesterol showed an increased occurrence of DM (*p*-value = 0.0002) and obesity (*p*-value < 0.0001), but not for hypertriglyceridemia (*p*-value = 0.4821), HBP (*p*-value = 0.1360), or hyperuricemia (*p*-value = 0.3756).

The presence of hypertriglyceridemia is associated with an increased occurrence of stroke (*p*-value = 0.0153), dementia (*p*-value = 0.0038), and cognitive impairment (*p*-value = 0.0021), but not for SLI (*p*-value = 0.7478) or PD (*p*-value = 0.3013). These patients presented significantly more DM (*p*-value < 0.0001), HBP (*p*-value = 0.0077), obesity (*p*-value < 0.0001), and hyperuricemia (*p*-value = 0.0466).

HBP associates, in these patients, significantly more dementia (*p*-value = 0.0107) but without influencing the presence of other NPSC. DM, hypo-HDL-cholesterolemia, obesity, hypertriglyceridemia, and hyperuricemia have statistical significance (*p*-value < 0.0001) and are more frequently present in patients with MetSy.

Finally, analysing the impact of the presence of obesity in these patients, we can see that there is no statistically significant difference between the two groups in regard to NPSC, even in the case of obese patients among the MetSy group who presented statistically significantly more DM, hypo-HDL-cholesterolemia, HBP, hypertriglyceridemia, and respectively hyperuricemia (all having a *p*-value < 0.0001).

Considering the above results, we analysed if the presence of MetSy may modify the survival without stroke probability. For that, we extracted the patients who had strokes prior to the AF diagnosis, and we found 994 patients from both groups. Subgroup 1 is comprised of patients who presented with a stroke prior to the diagnosis of AF. While subgroup 2 comprised 271 patients who presented with stroke after an AF diagnosis. According to these data, we found that patients in Group 1 develop stroke after 3.521 years. In Group 2, strokes occurred after approximately 3.286 years. Based on these findings, it can be stated that patients with AF and MetSy might be more likely to develop stroke later than those with AF but without MetSy (*p*-value = 0.0304).

The use of oral anticoagulation (OAC) was more common among Group 1 patients (65.2%) compared to Group 2 (57.7%) (*p*-value = 0.0293).

**Figure 2:**
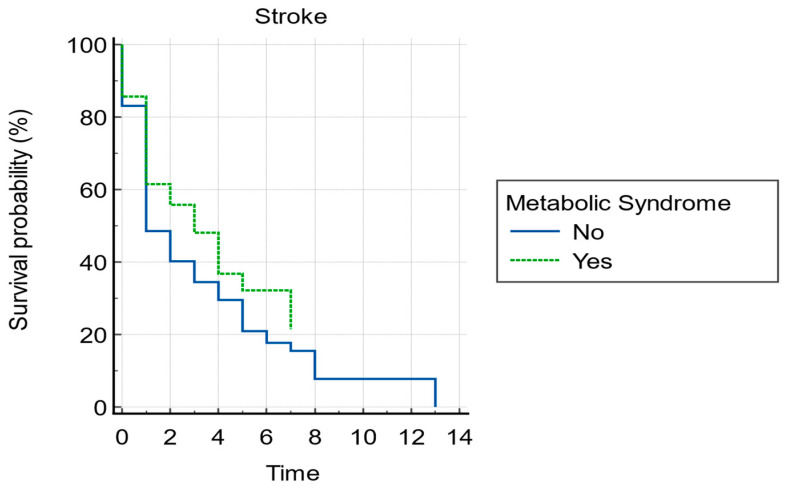
Kaplan-Maier survival curve without stroke for atrial fibrillation with or without metabolic syndrome.

**Figure 3:**
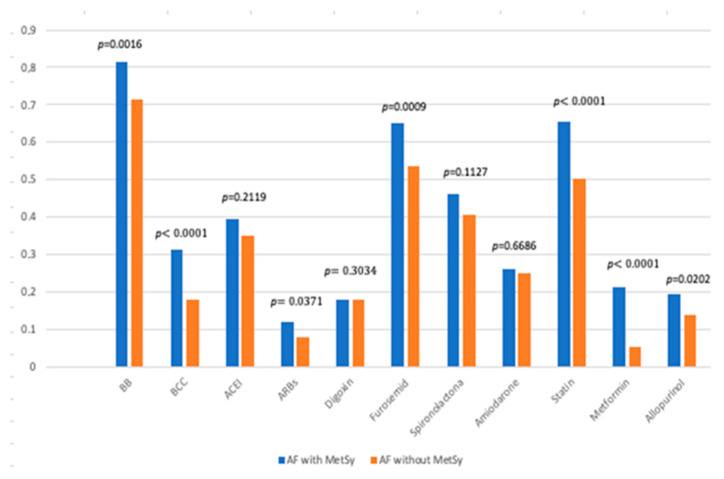
Drugs used in the treatment of study patients. AF—atrial fibrillation; MetSy—metabolic syndrome; BB—beta-blockers; CCB—calcium channel blockers; ACEI—angiotensin-converting enzyme inhibitors; ARBs—angiotensin receptor blockers; and p—level of statistical significance.

When we compared the other drugs used, statistical significance was noted for the use of beta-blockers (BB), calcium channel blockers (CCB), angiotensin receptor blockers (ARBs), furosemide, statins, metformin, and allopurinol (Figure 2 and Figure 3).

Analysing the impact of treatment regimen over the protective effect of the MetSy on the development risk of dementia and stroke, we found that in the case of dementia, the medication, even though proved to have a protective effect, was not seen to have statistical significance. Regarding stroke, our data showed an additional protective effect regarding its occurrence when patients were treated with digoxin (coefficient −0.34637, OR = 0.7072 with 95% CI [0.5063; 0.9879], *p*-value = 0.0422) and with BB (coefficient −0.51141, OR = 0.5996 with 95%CI [0.4401; 0.8170], *p*-value = 0.0012) and a supplementary risk if the MetSy was associated with ARBs (coefficient −0.5555, OR = 1.7429 with 95%CI [1.1142; 2.7265], *p*-value = 0.0150).

Regarding the mean hospitalisation period, longer stays were in favour of the patients with MetSy, with almost a day (+0.7795 day; *p*-value = 0.0142; 95% CI [0.1570; 1.4020]).

## 4. Discussion

The main finding of our study was that the metabolic syndrome might offer protection against the occurrence of dementia among patients with atrial fibrillation. Furthermore, we demonstrated that obesity offers a very important protective effect against other components of the metabolic syndrome. But further studies can confirm our findings.

The MetSy represents an association of factors whose presence is continuously on the rise. The reason behind this increase is correlated with the current lifestyle, characterized by an accentuated sedentarism. Early detection and interventions aimed at correcting these factors generally have a beneficial effect, reducing the occurrence of complications such as HBP, DM, dyslipidemia, stroke, and dementia.

In our study, the cumulative presence of HBP, DM, high triglyceride levels, and low HDL cholesterol levels led to a loss of statistical power in terms of the risk of AF (Table 3), even though it had a higher occurrence in the MetSy group. This situation accounts for the data showing an increase in the occurrence of AF correlated with increased triglyceride levels and decreased HDL-cholesterol, but also with the presence of DM and HBP [18]. However, it can be attributed to the protective effect conferred by the presence of obesity that is maintained regardless of the associated risk factors, which is another argument for the “paradoxical effect” of obesity.

Moreover, even if the presence of DM is associated with an increased occurrence of NPSC in our patients, we could not demonstrate significant differences in the presence of these complications even after individual analysis of these factors, with some minor exceptions. We believe that these aspects, for the groups analysed, are also due to the “protection” offered by the presence of obesity. However, as stated before, it is hard to draw conclusions without other studies reporting the same.

Even if advanced age is a risk factor for AF, stroke, and dementia (related or non-related to AF) [18], in the presence of MetSy, the age of the patients in our group is significantly lower.

The results obtained by analysing our groups of patients confirm the conclusions of the review published by Koutsonida et al. [19], which showed that the data of the analysed studies failed to demonstrate statistical significance regarding the association between the MetSy and the presence of cognitive impairment.

Furthermore, the presence of dementia in association with MetSy in the general population is inconsistently reported in studies, with some showing an increase in its occurrence in contrast to others that failed to demonstrate this association [20].

The risk of AF, as well as AF-related stroke, is reported to be increased by the presence of MetSy [18]. However, in the group analysed by us, the incidence of stroke between the two groups did not have a statistically significant difference. This behaviour can be based on the “paradoxes” reported for some of the component factors of MetSy.

The “Obesity Paradox” is not yet well documented, but it is once again demonstrated by the data obtained in our study, also confirming the presence of the “lean paradox” [21]. These results are in contrast to the fact that obesity represents an important risk factor for arrhythmias and is a striking feature that induces AF [22,23].

Furthermore, the “obesity paradox” is also present in terms of the survival of stroke patients, where obese patients have better survival rates one year after the first ischemic stroke compared to normal-weight patients [24]. In our study as well, the existence of a longer period until the appearance of the first ischemic stroke among patients with AF associated with MetSy compared to those without MetSy was documented.

There are studies that have shown an increase in the occurrence of dementia among middle-aged obese people [20]. In our study, obesity played a protective role against stroke and dementia.

Obesity, through the renin-angiotensin system (RAS), is involved in increasing sodium retention and increasing the release of adipokines, which are involved in the appearance of HBP [18]. An explanation of “the obesity paradox” is given by the importance of adipokines secreted by adipose tissue. These are involved in different endocrine mechanisms with pro- and anti-inflammatory effects [25]. One of the most studied is Adiponectin, which seems to offer some protection to the cardiovascular and metabolic systems as well as cerebral ischemia [26,27]. It seems that adiponectin is also involved in the modulation of the heart rate and the onset of AF [28]. More recent studies have highlighted the fact that this would provide a protective effect, including on the mortality rate of hospitalised patients [29].

The presence of hyperuricemia is frequently associated with HBP, DM type 2, AF, and MetSy [30]. Recent data show an association between the increased occurrence of neurodegenerative diseases (dementia, essential tremor, and PD) in patients with hyperuricemia (especially by decreasing regional and global brain volumes) [31]. In our study, hyperuricemia was present in a higher proportion of Group 1 patients (*p*-value < 0.0001), which necessitated the more frequent use of allopurinol as a therapy to lower serum uric acid levels. There are studies that have shown an improvement in insulin resistance as well as an improvement in the inflammatory syndrome under treatment with allopurinol [32]. There is also evidence demonstrating a decrease in the occurrence of stroke under treatment with allopurinol [33]. These data may justify, in part, the data presented in our study.

Increased serum angiotensin II levels are correlated with an increased occurrence of AF [34]. The involvement of renin-angiotensin system inhibitors in the prevention of stroke in patients with AF was also demonstrated by improving the function of the left atrial appendage (LAA) as well as by decreasing the risk of thrombus occurrence at the level of the LAA [35]. These drugs are involved in reducing the risk of AF [36] and even the onset of diabetes mellitus [37].

Limitations of the study: The major limitation of the study is its retrospective character. Apart from this, there is a lack of uniformity regarding the criteria addressed in the diagnosis of dementia, cognitive impairment, and PD because different neurologists, psychiatrists, and other specialists established these diagnoses, details of which were not collected. Furthermore, the lower age of the patients in Group 1 and the differences in the medications used in the treatment plans of the patients included in the study limit the study.

Further studies over an extended period of time with a larger cohort with standardised approaches and pre-set protocols for diagnosis, etc. would further help pave an accurate pathway towards setting new guidelines and helping obtain better outcomes. Evaluating new elements to improve the current therapeutic approach in patients presenting with considerable cardiovascular risk with the aim of preventing as effectively as possible the occurrence of neuro-psychological complications in these patients will further be beneficial both for the patients and the healthcare sector.

## 5. Conclusions

Based on the results obtained in our study, we can conclude that the metabolic syndrome might be helpful in offering some protection against the occurrence of dementia among atrial fibrillation patients but has no effect with regard to stroke. In regard to the appearance of dementia, MetSy might be labelled as a blessing in disguise. However, due to the lack of other studies on larger cohorts, it is hard to draw a concrete conclusion from our hypothesis presented in this manuscript regarding the positive effects of MetSy until the same is proven worldwide in other studies. Hence, further studies are required to gain a better understanding of the paradoxical effects of obesity. Early detection is key to preventing complications. Hence, timely evaluations and a multidisciplinary approach should be followed to evaluate the patients from a cardiac, neurological, psychiatric, and metabolic point of view.

## Figures and Tables

**Table 1:** Group characteristics (chi-squared test, except for age; student’s *t*-test unpaired).

Group	AF with MetSy 267 Patients	AF without MetSy 843 Patients	*p*-Value
Gender	M: 51.7% F: 48.3%	M: 47.8% F: 52.2%	0.8847
Mean age (years)	69.50 (95% CI) for the mean 68.3611 to 70.6501 Std. Dev. 9.49 years	73.94 95% CI for the mean 72.4875 to 73.9419 Std Dev 10.75 years	<0.0001
Place of residence	U: 47.9% R: 52.1%	U: 49.9% R:50.1%	<0.5463
COPD	25.1%	20.2%	0.0870
Asthma	13.5%	9.1%	<0.0406
HF	88.8%	77.8%	<0.0001
ICD	77.2%	63.7%	<0.0001
CKD stage > 3	63.3%	59.3%	0.2410
Hyperuricemia	22.8%	11.6%	<0.0001

AF—atrial fibrillation; MetSy—metabolic syndrome; M—male gender; F—female gender; U—urban (city); R—rural (village); COPD—chronic obstructive pulmonary disease; HF—heart failure; ICD—ischemic coronary disease; and CKD—chronic kidney disease.

**Table 2:** Neuropsychic changes in repartition (chi-squared test).

Group	AF and MetSy (267 Patients)	AF without MetSy (843 Patients)	*p*-Value
Stroke	37.5%	42.2%	0.1731
Dementia	4.5%	9.1%	<0.0001
SLI	7.1%	10.7%	0.0886
Cognitive impairment	10.9%	6%	0.0081
Parkinson’s disease	5.6%	5.0%	0.6818

AF—atrial fibrillation; MetSy—metabolic syndrome; and SLI—silent lacunar infarction.

**Table 3:** Logistic regression was used to analyse the effect of the presence of metabolic syndrome on the occurrence of dementia among atrial fibrillation patients.

Variable	Coefficient	Std. Error	Wald	*p*-Value	Odds Ratio	95% CI
HBP	0.043256	0.32026	0.01824	0.8926	1.0442	0.5574 to 1.9561
DM	0.36730	0.24558	2.2368	0.1348	1.4438	0.8922 to 2.3365
HyperTG	0.34083	0.27572	1.5280	0.2164	1.4061	0.8191 to 2.4139
HypoHDLc	0.25701	0.27859	0.8511	0.3562	1.2931	0.7490 to 2.2323
Obesity	−1.25852	0.35248	12.7487	0.0004	0.2841	0.1424 to 0.5668
Constant	−2.63705	0.30488	74.8141	<0.0001		

HBP—arterial hypertension; DM—diabetes mellitus; HyperTG—hypertriglyceridemia; and HypoHDLc—hypo high-density lipoprotein cholesterol.

## Data Availability

Not applicable.

## References

[1] Huang P.L. (2009). A comprehensive definition for metabolic syndrome. Dis. Model. Mech..

[2] Hindricks G., Potpara T., Dagres N., Arbelo E., Bax J.J., Blomström-Lundqvist C., Boriani G., Castella M., Dan G.A., Dilaveris P.E. 2020 ESC Guidelines for the Diagnosis and Management of Atrial Fibrillation Developed in Collaboration with the European Association for Cardio-Thoracic Surgery (EACTS): Supplementary Data. https://oup.silverchair-cdn.com/oup/backfile/Content_public/Journal/eurheartj/42/5/10.1093_eurheartj_ehaa612/3/ehaa612_supplementary_data.pdf?Expires=1629475752&Signature=kGhLjuMsKWF6KVV1fmivQpkdQ0LjMyn2epMoaoM8xkrrFh3OIIvdxl7oOxGnER-rRuN2H2ZhVTY1VK9j3HsZsYslznXMEtMnVJ~yxDg3pB3TLCzR5hr5fg6FilfLNmx82Vd5hXzF30woprzQ0zvFd5YYk8BdSzcUYI~2Fkfbh-wrISzNgoa~CrRqshNxm-fFBrBplTFItx-Q1OgrIbWxmTqP7cbI0KK5DR1qBBRBeqw-9mnaoJpz2xfGGeEiGCVPRVPpnH1HaNpSYogFMoecTpK~kYIH1yPSY4whr93TusgPy1ggm2Mxdsp846H4X9tGTgGTvBgO2W7q6mTwTLxnxw__&Key-Pair-Id=APKAIE5G5CRDK6RD3PGA.

[3] Chugh S.S., Havmoeller R., Narayanan K., Singh D., Rienstra M., Benjamin E.J., Gillum R.F., Kim Y.H., McAnulty J.H., Zheng Z.J. (2014). Worldwide epidemiology of atrial fibrillation: A Global Burden of Disease 2010 Study. Circulation.

[4] Hindricks G., Potpara T., Dagres N., Arbelo E., Bax J.J., Blomström-Lundqvist C., Boriani G., Castella M., Dan G.-A., E Dilaveris P. (2020). 2020 ESC Guidelines for the diagnosis and management of atrial fibrillation developed in collaboration with the European Association for Cardio-Thoracic Surgery (EACTS): The Task Force for the diagnosis and management of atrial fibrillation of the European Society of Cardiology (ESC) Developed with the special contribution of the European Heart Rhythm Association (EHRA) of the ESC. Eur. Heart J..

[5] Tai W.A. (2022). Stroke: Primary Prevention. FP Essent.

[6] Hachinski V., Einhäupl K., Ganten D., Alladi S., Brayne C., Stephan B.C., Sweeney M.D., Zlokovic B., Iturria-Medina Y., Iadecola C. (2019). Preventing dementia by preventing stroke: The Berlin Manifesto. Alzheimer’s Dement..

[7] The IDF Consensus Worldwide Definition of the Metabolic Syndrome. 2006–2020. https://idf.org/our-activities/advocacy-awareness/resources-and-tools/60:idfconsensus-worldwide-definitionof-the-metabolic-syndrome.html.

[8] Tahmi M., Palta P., Luchsinger J.A. (2021). Metabolic Syndrome and Cognitive Function. Curr. Cardiol. Rep..

[9] Kirvalidze M., Hodkinson A., Storman D., Fairchild T.J., Bała M.M., Beridze G., Zuriaga A., Brudasca N.I., Brini S. (2022). The role of glucose in cognition, risk of dementia, and related biomarkers in individuals without type 2 diabetes mellitus or the metabolic syndrome: A systematic review of observational studies. Neurosci. Biobehav. Rev..

[10] Koliaki C., Liatis S., Kokkinos A. (2019). Obesity and cardiovascular disease: Revisiting an old relationship. Metabolism.

[11] Luo L., Yang M., Hao Q., Yue J., Dong B. (2013). Cross-Sectional Study Examining the Association between Metabolic Syndrome and Cognitive Function among the Oldest Old. J. Am. Med. Dir. Assoc..

[12] Fuchs F.D., Whelton P.K. (2020). High Blood Pressure and Cardiovascular Disease. Hypertension.

[13] Komulainen P., Lakka T.A., Kivipelto M., Hassinen M., Helkala E.-L., Haapala I., Nissinen A., Rauramaa R. (2007). Metabolic Syndrome and Cognitive Function: A Population-Based Follow-up Study in Elderly Women. Dement. Geriatr. Cogn. Disord..

[14] Ivanova N., Liu Q., Agca C., Agca Y., Noble E.G., Whitehead S.N., Cechetto D.F. (2020). White matter inflammation and cognitive function in a co-morbid metabolic syndrome and prodromal Alzheimer’s disease rat model. J. Neuroinflamm..

[15] Suzuki S. (2011). “Cholesterol Paradox” in Atrial Fibrillation. Circ. J..

[16] Alonso A., Yin X., Roetker N.S., Magnani J.W., Kronmal R.A., Ellinor P.T., Chen L.Y., Lubitz S.A., McClelland R.L., McManus D.D. (2014). Blood Lipids and the Incidence of Atrial Fibrillation: The Multi-Ethnic Study of Atherosclerosis and the Framingham Heart Study. J. Am. Heart Assoc..

[17] (2012). KDIGO 2012 Clinical Practice Guideline for the Evaluation and Management of Chronic Kidney Disease. https://kdigo.org/wp-content/uploads/2017/02/KDIGO_2012_CKD_GL.pdf.

[18] Hajhosseiny R., Matthews G.K., Lip G.Y. (2015). Metabolic syndrome, atrial fibrillation, and stroke: Tackling an emerging epidemic. Heart Rhythm..

[19] Koutsonida M., Markozannes G., Bouras E., Aretouli E., Tsilidis K.K. (2022). Metabolic syndrome and cognition: A systematic review across cognitive domains and a bibliometric analysis. Front. Psychol..

[20] Borshchev Y.Y., Uspensky Y.P., Galagudza M.M. (2019). Pathogenetic pathways of cognitive dysfunction and dementia in metabolic syndrome. Life Sci..

[21] Powell-Wiley T.M., Poirier P., Burke L.E., Després J.-P., Gordon-Larsen P., Lavie C.J., Lear S.A., Ndumele C.E., Neeland I.J., Sanders P. (2021). Obesity and Cardiovascular Disease: A Scientific Statement from the American Heart Association. Circulation.

[22] Pathak R.K., Mahajan R., Lau D.H., Sanders P. (2015). The Implications of Obesity for Cardiac Arrhythmia Mechanisms and Management. Can. J. Cardiol..

[23] Lavie C.J., Pandey A., Lau D.H., Alpert M.A., Sanders P. (2017). Obesity and Atrial Fibrillation Prevalence, Pathogenesis, and Prognosis: Effects of Weight Loss and Exercise. J. Am. Coll. Cardiol..

[24] Chaudhary D., Khan A., Gupta M., Hu Y., Li J., Abedi V., Zand R. (2021). Obesity and mortality after the first ischemic stroke: Is obesity paradox real?. PLoS ONE.

[25] Feijóo-Bandín S., Aragón-Herrera A., Moraña-Fernández S., Anido-Varela L., Tarazón E., Roselló-Lletí E., Portolés M., Moscoso I., Gualillo O., González-Juanatey J.R. (2020). Adipokines and Inflammation: Focus on Cardiovascular Diseases. Int. J. Mol. Sci..

[26] Menzaghi C., Trischitta V. (2018). The Adiponectin Paradox for All-Cause and Cardiovascular Mortality. Diabetes.

[27] Wu M.-H. (2019). Adiponectin: A pivotal role in the protection against cerebral ischemic injury. Neuroimmunol. Neuroinflamm..

[28] Zhu T., Chen M., Wang M., Wang Z., Wang S., Hu H., Ma K., Jiang H. (2022). Association between adiponectin-to-leptin ratio and heart rate variability in new-onset paroxysmal atrial fibrillation: A retrospective cohort study. Ann. Noninvasive Electrocardiol..

[29] Hindsberger B., Lindegaard B., Andersen L.R., Israelsen S.B., Pedersen L., Szecsi P.B., Benfield T. (2023). Circulating Adiponectin Levels Are Inversely Associated with Mortality and Respiratory Failure in Patients Hospitalized with COVID-19. Int. J. Endocrinol..

[30] Borghi C., Agabiti-Rosei E., Johnson R.J., Kielstein J.T., Lurbe E., Mancia G., Redon J., Stack A.G., Tsioufis K.P. (2020). Hyperuricaemia and gout in cardiovascular, metabolic and kidney disease. Eur. J. Intern. Med..

[31] Topiwala A., Mankia K., Bell S., Webb A., Ebmeier K.P., Howard I., Wang C., Alfaro-Almagro F., Miller K., Burgess S. (2023). Association of gout with brain reserve and vulnerability to neurodegenerative disease. Nat. Commun..

[32] Takir M., Kostek O., Ozkok A., Elcioglu O.C., Bakan A., Erek A., Mutlu H.H., Telci O., Semerci A., Odabas A.R. (2015). Lowering Uric Acid with Allopurinol Improves Insulin Resistance and Systemic Inflammation in Asymptomatic Hyperuricemia. J. Investig. Med..

[33] MacIsaac R.L., Salatzki J., Higgins P., Walters M.R., Padmanabhan S., Dominiczak A.F., Touyz R.M., Dawson J. (2016). Allopurinol and Cardiovascular Outcomes in Adults with Hypertension. Hypertension.

[34] Demers J., Ton A., Huynh F., Thibault S., Ducharme A., Paradis P., Nemer M., Fiset C. (2022). Atrial Electrical Remodeling in Mice with Cardiac-Specific Overexpression of Angiotensin II Type 1 Receptor. J. Am. Heart Assoc..

[35] Suo Y., Zhang Y., Wang Y., Yuan M., Kariyawasam S., Tse G., Liu T., Fu H., Li G. (2018). Renin–angiotensin system inhibition is associated with reduced risk of left atrial appendage thrombosis formation in patients with atrial fibrillation. Cardiol. J..

[36] Schneider M.P., Hua T.A., Böhm M., Wachtell K., Kjeldsen S.E., Schmieder R.E. (2010). Prevention of Atrial Fibrillation by Renin-Angiotensin System Inhibition: A Meta-Analysis. J. Am. Coll. Cardiol..

[37] Gherasim L. (2022). Association of Atrial Fibrillation with Diabetes Mellitus, High Risk Comorbidities. Maedica.

